# *N*′-[(1*E*)-(5-Nitro­furan-2-yl)methyl­idene]thio­phene-2-carbohydrazide: crystal structure and Hirshfeld surface analysis

**DOI:** 10.1107/S2056989016009968

**Published:** 2016-06-24

**Authors:** Laura N. F. Cardoso, Thais C. M. Nogueira, James L. Wardell, Solange M. S. V. Wardell, Marcus V. N. de Souza, Mukesh M. Jotani, Edward R. T. Tiekink

**Affiliations:** a Fundaçaö Oswaldo Cruz, Instituto de Tecnologia em Fármacos-Far Manguinhos, 21041-250 Rio de Janeiro, RJ, Brazil; b Department of Chemistry, University of Aberdeen, Old Aberdeen, AB24 3UE, Scotland; c CHEMSOL, 1 Harcourt Road, Aberdeen AB15 5NY, Scotland; d Department of Physics, Bhavan’s Sheth R. A. College of Science, Ahmedabad, Gujarat 380 001, India; e Research Centre for Crystalline Materials, Faculty of Science and Technology, Sunway University, 47500 Bandar Sunway, Selangor Darul Ehsan, Malaysia

**Keywords:** crystal structure, carbohydrazide, hydrogen bonding, conformation, Hirshfeld surface analysis

## Abstract

In the title carbohydrazide, C_10_H_7_N_3_O_4_S, the dihedral angle between the terminal five-membered rings is 27.4 (2)°, with these lying to the same side of the plane through the central CN_2_C(=O) atoms (r.m.s. deviation = 0.0403 Å), leading to a curved mol­ecule. The conformation about the C=N imine bond [1.281 (5) Å] is *E*, and the carbonyl O and amide H atoms are *anti*. In the crystal, N—H⋯O hydrogen bonds lead to supra­molecular chains, generated by a 4_1_ screw-axis along the *c* direction. A three-dimensional architecture is consolidated by thienyl-C—H⋯O(nitro) and furanyl-C—H⋯O(nitro) inter­actions, as well as π–π inter­actions between the thienyl and furanyl rings [inter-centroid distance = 3.515 (2) Å]. These, and other, weak inter­molecular inter­actions, *e.g*. nitro-N—O⋯π(thien­yl), have been investigated by Hirshfeld surface analysis, which confirms the dominance of the conventional N—H⋯O hydrogen bonding to the overall mol­ecular packing.

## Abstract 2

The title mol­ecule is curved as seen in the dihedral angle [27.4 (2)°] between the outer rings. Supra­molecular chains about a 4_1_ screw axis are formed by amide-N—H⋯O(carbon­yl) hydrogen bonding.

## Chemical context   

Thio­phene and its derivatives have been well studied as materials, *e.g*. in applications in organic electronics and photonics (Perepichka & Perepichka, 2009 ▸) and in the medical area. In the latter context, the thio­phene nucleus is present in many natural and synthetic products having a wide range of pharmacological activities, such as anti-viral (Chan *et al.*, 2004 ▸), anti-cancer (Romagnoli *et al.*, 2011 ▸), anti-bacterial (Sivadas *et al.*, 2011 ▸; Jain *et al.*, 2012 ▸), anti-fungal (Jain *et al.*, 2012 ▸; Saeed *et al.*, 2010 ▸), anti-inflammatory (Kumar *et al.*, 2004 ▸) and anti-microbial and anti-tuberculosis (anti-TB) activities (Abdel-Aal *et al.*, 2010 ▸). Our inter­ests in the biological activities and structural chemistry of heterocyclic compounds have led us to investigate thio­phene and its derivatives as tuberculostatic agents. Thus, some of us have reported the anti-TB activities of acetamido derivatives, 2-(*RR*′NCOCH_2_)-thio­phene (Lourenço *et al.*, 2007 ▸; de Sousa, Ferreira *et al.*, 2008 ▸; de Sousa, Lourenço *et al.*, 2008 ▸), aceto­hydrazide derivatives 2-(ArCH=N–N*R*COCH_2_)-thio­phene, **1** (Cardoso *et al.*, 2014 ▸; Cardoso *et al.*, 2016*a* ▸) and 2-(ArCH=N–N*R*CO)-thio­phene, **2**, *R* = H or Me (Cardoso *et al.*, 2016*a* ▸). Herein, we wish to report the crystal structure of the title compound, (*E*)-*N*′-(5-nitro­furan-2-yl­methyl­ene)thio­phene-2-carbohydrazide, (I), Scheme 1, as well as an analysis of its Hirshfeld surface. Crystal structures of **1**: Ar = 5-nitro­thien-2-yl; *R* = H, Me (Cardoso *et al.*, 2016*b* ▸), **2**: Ar = 5-nitro­thien-2-yl; *R* = H Me (Cardoso *et al.*, 2016*b* ▸) and **1**: Ar = 5-HOC_6_H_4_: *R* = H (Cardoso *et al.*, 2014 ▸) have been previously published.
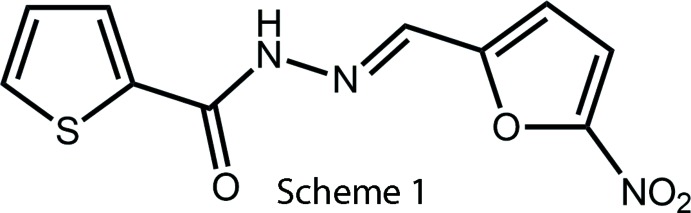


## Structural commentary   

**Figure 1:**
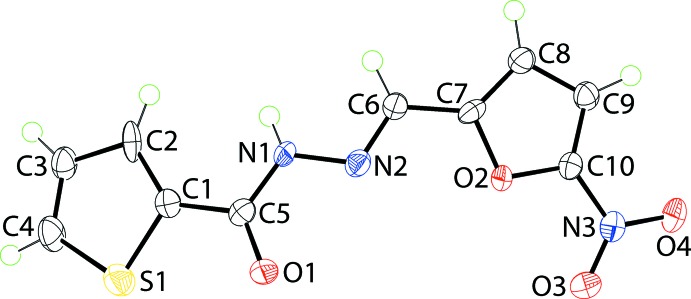
The mol­ecular structure of (I), showing displacement ellipsoids at the 70% probability level.

In (I), Fig. 1 ▸, the conformation about the C6=N2 bond [1.281 (5) Å] is *E*. A 5-nitro­furan-2-yl ring is connected at the C6 atom. The furanyl ring is almost planar [r.m.s deviation = 0.006 Å] and the nitro group is almost co-planar with its attached ring as seen in the O3—N3—C10—O2 torsion angle of −1.7 (5)°. The thienyl ring is also planar within experimental error [r.m.s. deviation = 0.005 Å] and orientated so that the sulfur atom is *syn* to the carbonyl-O1 atom. Overall, the mol­ecule is curved with the rings lying to the same side of the plane through the bridging CN_2_C(=O) atoms, r.m.s. deviation = 0.0403 Å, with twists noted in both the S1—C1—C5—O1 and N2—C6—C7—O2 torsion angles of −9.8 (5) and 5.4 (6)°, respectively; the dihedral angle between the five-membered rings is 27.4 (2)°.

## Supra­molecular features   

**Figure 2:**
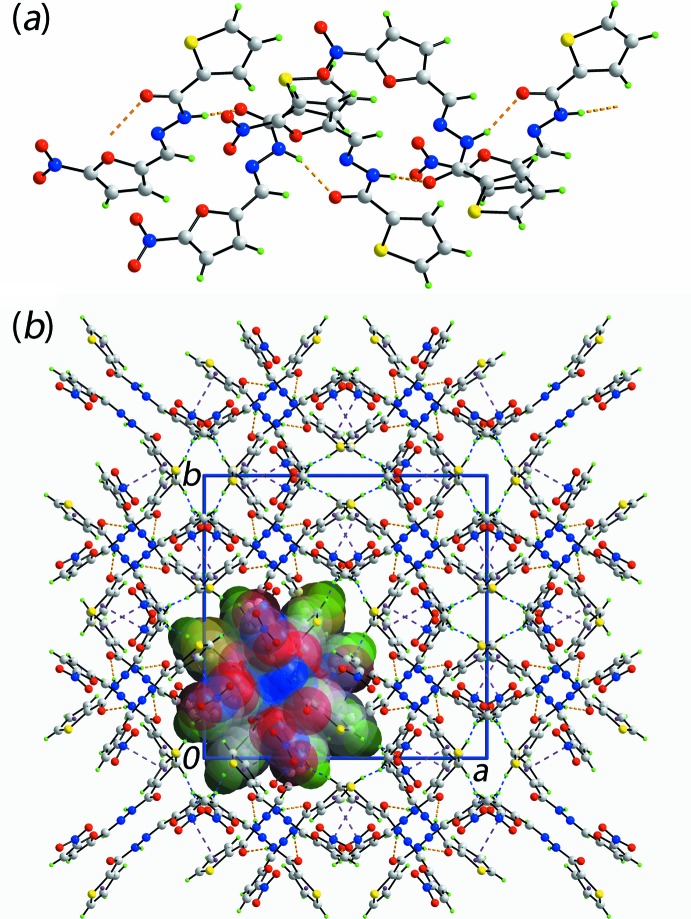
The mol­ecular packing in (I), showing (*a*) a view of a supra­molecular chain aligned along the *c* axis sustained by amide-*N*—*H*⋯*O*(carbon­yl) hydrogen bonds and (*b*) a view in projection down the *c* axis of the unit-cell contents; one chain has been highlighted in space-filling mode. The N—H⋯O, C—H⋯O and π–π inter­actions are shown as orange, blue and purple dashed lines, respectively. Colour code: S yellow, O red, N blue, C grey and H green.

The *anti* relationship between the carbonyl-O and amide-H atoms enables the formation of directional N—H⋯O hydrogen bonds leading to supra­molecular chains, generated by a 4_1_ screw-axis propagating along the *c*-axis direction, Fig. 2 ▸ *a* and Table 1 ▸. The chains are connected into a three-dimensional architecture by thienyl-C—H⋯O(nitro) and furanyl-C—H⋯O(nitro) inter­actions, involving the same nitro-O4 atom, Table 1 ▸. In addition, π–π inter­actions are formed between the two five-membered rings with the inter-centroid distance being 3.515 (2) Å, and the angle of inclination is 3.9 (2)° for symmetry operation: (i) 1 − *y*, 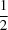 − *x*, −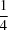 + *z*. A view of the unit-cell contents is shown in Fig. 2 ▸ *b*.

## Hirshfeld surface analysis   

*Crystal Explorer 3.1* (Wolff *et al.*, 2012 ▸) was used to generate Hirshfeld surfaces mapped over *d*_norm_, *d*_e_, shape-index, curvedness and electrostatic potential. The latter were calculated using *TONTO* (Spackman *et al.*, 2008 ▸; Jayatilaka *et al.*, 2005 ▸) integrated into *Crystal Explorer*, wherein the experimental structure was used as the input geometry. In addition, the electrostatic potentials were mapped on Hirshfeld surfaces using the STO-3G basis set at Hartree–Fock level of theory over a range ±0.12 au. The contact distances *d*_i_ and *d*_e_ from the Hirshfeld surface to the nearest atom inside and outside, respectively, enable the analysis of inter­molecular inter­actions through the mapping of *d*_norm_. The combination of *d*_e_ and *d*_i_ in the form of a two-dimensional fingerprint plot (McKinnon *et al.*, 2004 ▸) provides a useful summary of inter­molecular contacts in the crystal.

**Figure 3:**
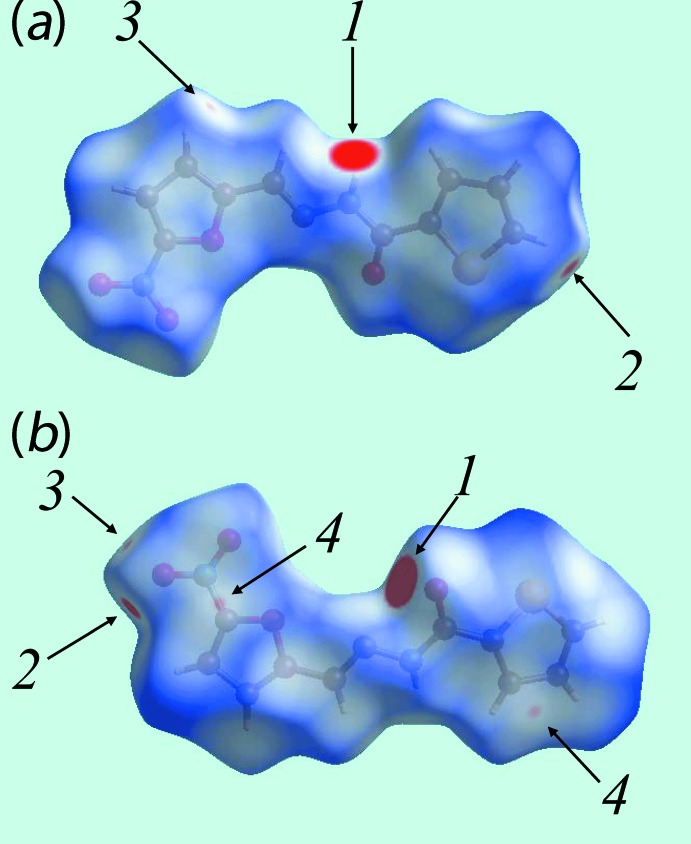
Two views of the Hirshfeld surface mapped over *d*_norm_ for (I), with labels 1, 2, 3 and 4 indicating specific inter­molecular inter­actions discussed in the text.

**Figure 4:**
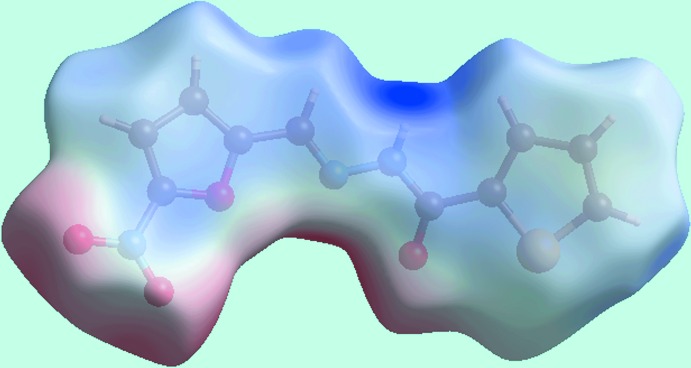
A view of the Hirshfeld surface mapped over electrostatic potential for (I). The red and blue regions represent negative and positive electrostatic potentials, respectively.

**Figure 5:**
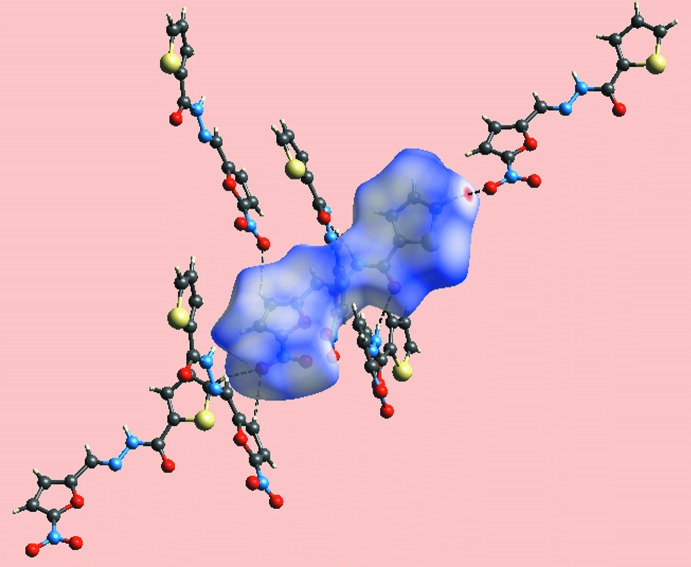
A view of Hirshfeld surface mapped over *d*_norm_ for showing inter­molecular inter­actions about a reference mol­ecule of (I).

Two views of Hirshfeld surfaces calculated for (I), mapped over *d*_norm_ in the −0.1 to 1.2 Å range are shown in Fig. 3 ▸. The bright-red spots near the amino-N—H and carbonyl-O atoms, labelled as ‘1’ in Fig. 3 ▸, indicate their roles as respective donor and acceptor sites in the dominant N—H⋯O hydrogen bonding in the crystal. These also appear as blue and red regions, respectively, corresponding to positive and negative electrostatic potentials, respectively, on the Hirshfeld surface mapped over electrostatic potential in Fig. 4 ▸. The light-red spots labelled as ‘2’ and ‘3’ in Fig. 3 ▸, and light-blue and light-red regions in Fig. 4 ▸, represent the inter­molecular thienyl-C—H⋯O(nitro) and furanyl-C—H⋯O(nitro) inter­actions involving the nitro-O4 atom as described above in *Supra­molecular features*. The immediate environment about the mol­ecule within *d*_norm_ mapped Hirshfeld surface mediated by the above inter­actions is illustrated in Fig. 5 ▸.

The presence of a short inter­molecular C⋯C contact between thienyl-C2 and furanyl-C10 atoms, Table 2 ▸, which fall within π–π contact between the thienyl and furanyl rings can also be viewed as faint-red spots near these atoms, labelled as ‘4’ in Fig. 3 ▸. In the crystal, a comparatively weak N—O⋯π inter­action (Spek, 2009 ▸) between the nitro—O4 atom and a symmetry-related thienyl ring [N3⋯*Cg*(S1,C1–C4) = 3.506 (4) Å, O4⋯*Cg*(S1,C1–C4) = 3.639 (4) Å and N3—O4⋯*Cg* = 74.0 (2)°] is also evident from the light-blue and red regions corresponding to their respective potentials on the Hirshfeld surface mapped over electrostatic potential in Fig. 4 ▸.

**Figure 6:**
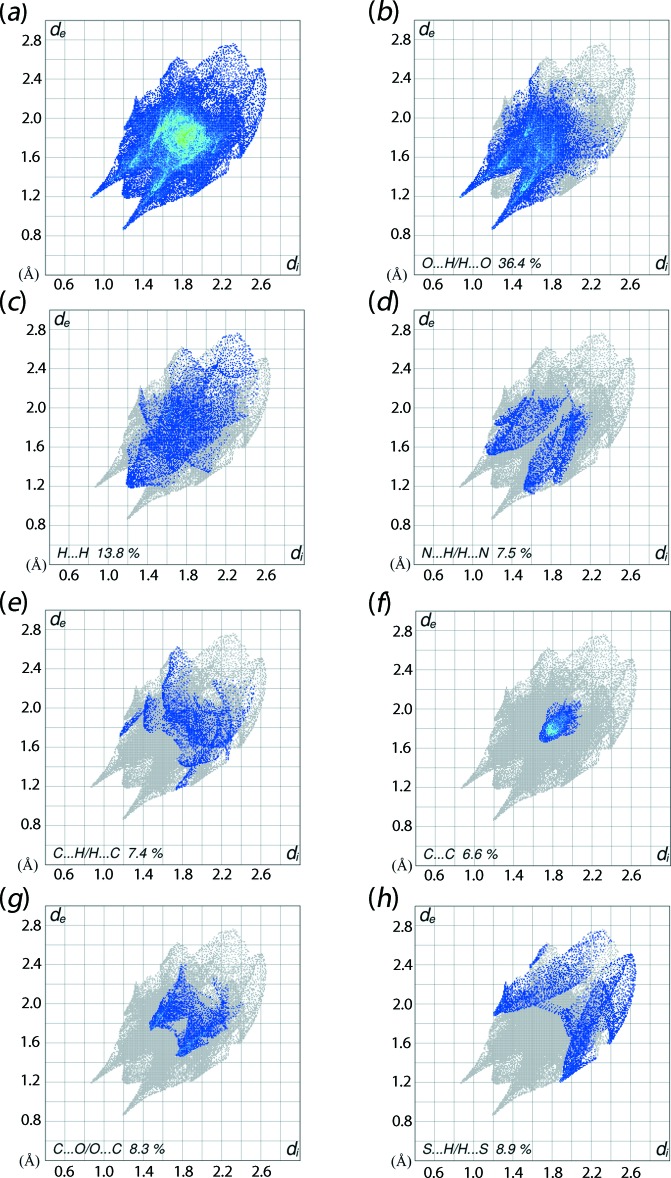
The two-dimensional fingerprint plots for (I), showing (*a*) all inter­actions, and delineated into (*b*) O⋯H/H⋯O, (*c*) H⋯H, (*d*) N⋯H/H⋯N, (*e*) C⋯H/H⋯C, (*f*) C⋯C, (*g*) C⋯O/H⋯O and (*h*) S⋯H/H⋯S inter­actions.

**Figure 7:**
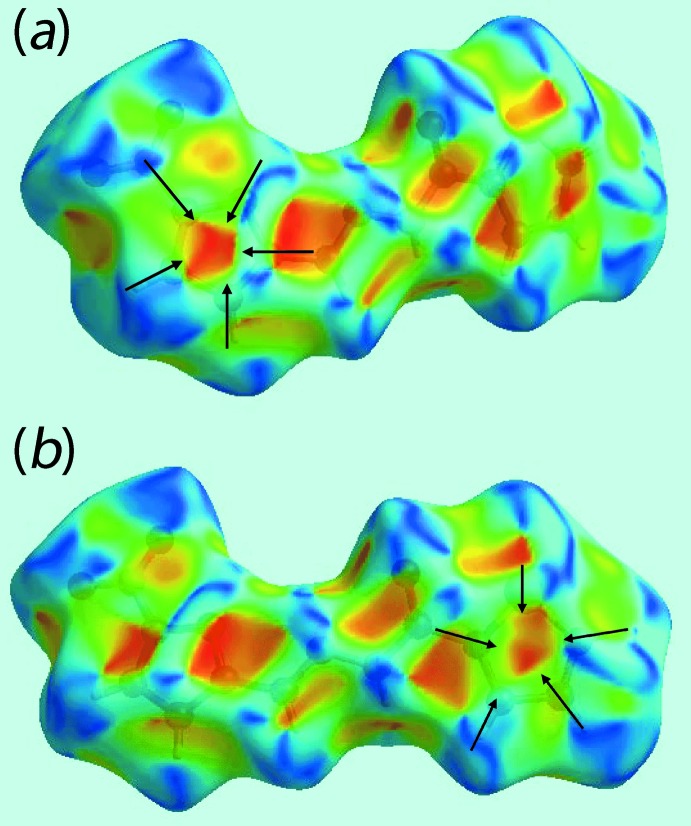
Two views of Hirshfeld surface mapped with shape-index property for (I). The pairs of red and blue regions identified with arrows indicate π–π stacking inter­actions.

**Figure 8:**
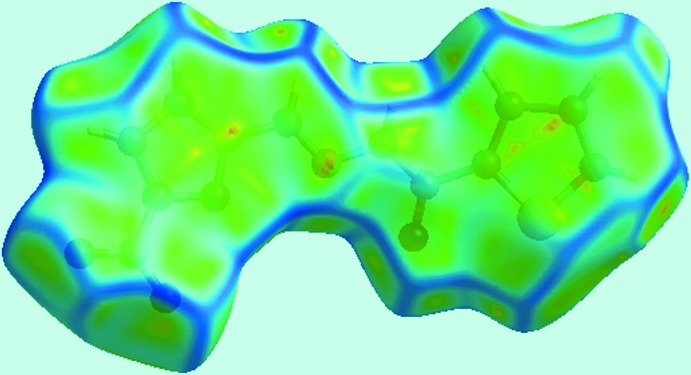
A view of Hirshfeld surface mapped over curvedness for (I). The flat regions highlight the involvement of rings in π–π stacking inter­actions.

The overall two-dimensional fingerprint plot is shown in Fig. 6 ▸ *a* and those delineated into O⋯H/H⋯O, H⋯H, N⋯H/H⋯N, C⋯H/H⋯C, C⋯C, C⋯O/O⋯C and S⋯H/H⋯S contacts (McKinnon *et al.*, 2007 ▸) are illustrated in Fig. 6 ▸ *b*–*h*, respectively; their relative contributions to the overall Hirshfeld surface are summarized in Table 3 ▸. In the fingerprint plot delineated into O⋯H/H⋯O contacts, which make the greatest contribution to the Hirshfeld surface, *i.e*. 36.4%, arises from the N—H⋯O hydrogen bond and is viewed as a pair of spikes with tips at *d*_e_ + *d*_i_ ∼2.1 Å in Fig. 6*b* ▸. The C—H⋯O inter­actions, which are masked by the above inter­actions, appear as the groups of green points appearing in pairs in the plot. However, a forceps-like distribution of points in the fingerprint plot delineated into C⋯O/O⋯C contacts, Fig. 6 ▸ *g*, with the tips at *d*_e_ + *d*_i_ ∼2.3 Å is indicative of C—H⋯O inter­actions. In the fingerprint plot corresponding to H⋯H contacts, which make the next most significant contribution to the surface, Fig. 6 ▸ *c*, the points are scattered in the plot at (*d*_e_, *d*_i_) distances greater than their van der Waals separations with the comparatively low contribution, *i.e*. 13.6%, due to the relatively low hydrogen-atom content in the mol­ecule. The absence of characteristic wings in the fingerprint plot delineated into C⋯H/H⋯C and the low contribution to the Hirshfeld surface, Fig. 6 ▸ *e* and Table 3 ▸, clearly indicate the absence of C—H⋯π inter­actions in the crystal. However, a pair of thin edges with their ends at *d*_e_ + *d*_i_ ∼2.9 Å belong to short inter­atomic C⋯H contacts, Table 2 ▸. The lung-shaped distribution of points with the bending at at *d*_e_ + *d*_i_ ∼2.7 Å in the fingerprint plot corresponding to N⋯H/H⋯N contacts, Fig. 6 ▸ *e*, with a 7.5% contribution to the Hirshfeld surface is the result of short inter­atomic N⋯H/H⋯N contacts, Table 2 ▸. The C⋯C contacts assigned to the short C2⋯C10 contact and π–π stacking inter­actions appear as the distribution of points around *d*_e_ = *d*_i_ ∼1.7 Å, Fig. 6 ▸ *f*. The presence of π–π stacking inter­actions between the symmetry-related thienyl and furanyl rings is also indicated by the appearance of red and blue triangle pairs on the Hirshfeld surface mapped with the shape-index property identified with arrows in the images of Fig. 7 ▸, and in the flat region on the Hirshfeld surface mapped over curvedness in Fig. 8 ▸. Finally, although the S⋯H/H⋯S contacts in the structure of (I) make a 8.9% contribution to the surface, and also show a nearly symmetrical distribution of points in the corresponding fingerprint plot, Fig. 6 ▸ *h*, they do not have a significant influence on the mol­ecular packing as they are separated at distances greater than the sum of their van der Waals radii.

The final analysis based on the Hirshfeld surfaces is an evaluation of enrichment ratios (ER) (Jelsch *et al.*, 2014 ▸); a list of the ER values is given in Table 4 ▸. The low content of hydrogen in the mol­ecular structure of (I) yields a very low ER, 0.72, indicating no propensity to form inter­molecular H⋯H contacts. The ER value of 1.55 from O⋯H/H⋯O contacts is in the expected 1.2–1.6 range and confirm their involvement in the N—H⋯O and C—H⋯O inter­actions. The presence of inter­molecular C—H⋯O inter­actions is also confirmed through the ER value near to unity *i.e*. 0.99, corresponding to the C⋯O/O⋯C contacts. The high propensity to form π–π stacking inter­actions between the thienyl and furanyl rings is reflected from the high enrichment ratio 2.66 for C⋯C contacts. The ER value of 1.26 resulting from 6.75% of the surface occupied by nitro­gen atoms and a 7.5% contribution to the Hirshfeld surface from N⋯H/H⋯N contacts is due to the presence of short N⋯H contacts in the structure, Table 2 ▸. The ER values < 1 related to other contacts and low % contribution to the surface indicate their low significance in the crystal.

## Database survey   

A search of the crystallographic literature (Groom *et al.*, 2016 ▸) reveals one closely related structure, namely the species with a methyl group rather than a nitro group, *N*′-[(5-methyl-2-fur­yl)methyl­ene]thio­phene-2-carbohydrazide [(II); Jiang, 2010 ▸].
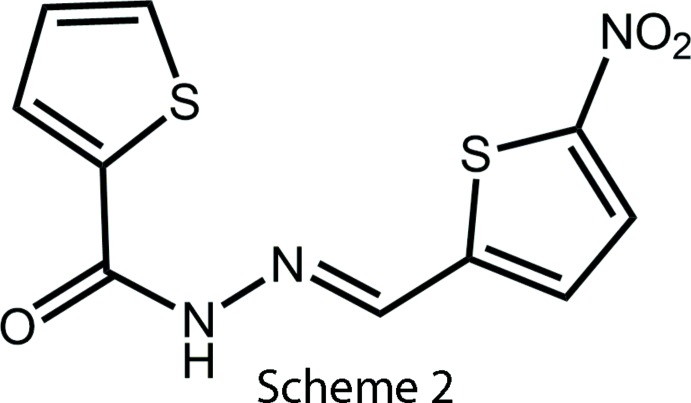


**Figure 9:**
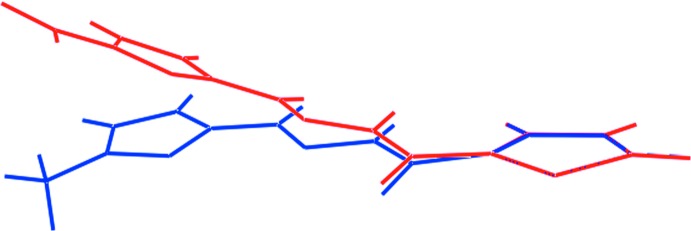
Overlay diagram of mol­ecules of (I) (red image) and (II) (blue). The mol­ecules have been overlapped so that the five-membered rings are coincident.

The relative dispositions of the heteroatoms in the two structures are the same but, the twist in (II) is significantly less as seen in the dihedral angle of 10.2 (6)° between the five-membered rings. This is highlighted in the overlay diagram in Fig. 9 ▸. The mol­ecular structure of the all thienyl analogue of (I) has been described recently (Cardoso *et al.*, 2016*b* ▸). There are two almost identical, near planar mol­ecules in the asymmetric unit and each adopts the conformation indicated in Scheme 2, which might be described as having the thienyl-S atoms *syn*. The intra­molecular S⋯S separations of 3.770 (4) and 3.879 (4) Å, are beyond the sum of their van der Waals radii. The conformational differences found for the thienyl mol­ecules is consistent with our NMR studies that indicate multiple conformations exist in solution for these compounds.

## Synthesis and crystallization   

**Figure 10:**
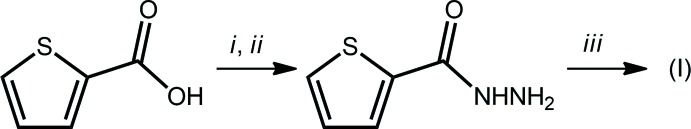
Preparation of the title compound. Reagents: *i* = SO_2_Cl_2_, MeOH; *ii* = N_2_H_2_·H_2_O, EtOH; *iii* = 5-nitro­furan­carbaldehyde, EtOH.

The title compound was prepared following a procedure outlined in Fig. 10 ▸. Yellow rods of (I) were grown by slow evaporation of a methanol solution held at room temperature. Yellow solid; m.p.: 528–529 K. IR ν_max_ (cm^−1^; KBr disc): 1629 (C=O); 3209 (N—H). ^1^H NMR (400 MHz; DMSO) δ: 12.26 (1H; NH), 8.10–7.96 (3H; *m*; H-4′; H-8′ and H-9′), 7.81 (1H; *d*; *J*_HH_ = 3.9 Hz; H-5), 7.28 (1H; *d*; *J*_HH_ = 3.9 Hz; H-4), 7.26-7.24 (1H; *m*; H-3). ^13^C NMR (100 MHz DMSO) δ: 161.6 (C=O), 157.9 (C-2), 151.6 (C-4′), 137.5 (C-5′), 135.2 (C-3), 132.7 (C-2) 131.4 (C-7′), 129.7 (C-8′), 128.2 (C-9′), 127.1 (C-9′). HRMS *m*/*z*: 288.0082 [*M* + Na]^+^; (calculated for [C_10_H_7_N_3_O_4_S+Na]^+^: 288.0055.

## Refinement details   

Crystal data, data collection and structure refinement details are summarized in Table 5 ▸. The C-bound H atoms were geometrically placed (C—H = 0.95 Å) and refined as riding with *U*_iso_(H) = 1.2*U*_eq_(C). The N-bound H atom was located from a difference map and refined with (N—H = 0.88±0.01 Å), and with *U*_iso_(H) = 1.2*U*_eq_(C). The slightly elongated displacement ellipsoid for the C2 atom in the thienyl ring is likely due to unresolved disorder in the ring where the second, co-planar orientation related by 180° to that modelled is present. However, this was not modelled as the maximum residual electron density peak was only 0.46 e Å^−3^, 0.61 Å from the C2 atom. It is also noted that the relevant S—C and C—C bond lengths show the expected values.

## Supplementary Material

Crystal structure: contains datablock(s) I, global. DOI: 10.1107/S2056989016009968/hb7593sup1.cif

Structure factors: contains datablock(s) I. DOI: 10.1107/S2056989016009968/hb7593Isup2.hkl

Supporting information file. DOI: 10.1107/S2056989016009968/hb7593Isup3.cml

CCDC reference: 1486459

Additional supporting information: crystallographic information; 3D view; checkCIF report

## Figures and Tables

**Table 1:** Hydrogen-bond geometry (Å, °)

*D*—H⋯*A*	*D*—H	H⋯*A*	*D*⋯*A*	*D*—H⋯*A*
N1—H1*N*⋯O1^i^	0.87 (3)	2.05 (3)	2.882 (4)	159 (3)
C4—H4⋯O4^ii^	0.95	2.42	3.293 (6)	152
C8—H8⋯O4^iii^	0.95	2.53	3.242 (5)	132

Symmetry codes: (i) 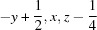; (ii) 
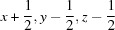
; (iii) 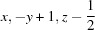.

**Table 2:** Summary of short inter­atomic contacts (Å) in the crystal of the title compound

Contact	Distance	Symmetry operation
C2⋯C10	3.361 (5)	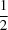 − *x*, 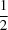 − *y*, − 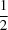 + *z*
C5⋯H2	2.89	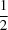 − *x*, *y*, 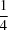 + *z*
N2⋯H6	2.72	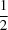 − *x*, *y*, 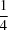 + *z*
N2⋯H1*N*	2.69 (4)	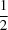 − *x*, *y*, 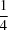 + *z*
O1⋯H2	2.68	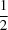 − *x*, *y*, 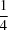 + *z*
O1⋯H6	2.68	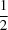 − *x*, *y*, 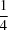 + *z*

**Table 3:** Percentage contribution of the different inter­molecular inter­actions to the Hirshfeld surface of the title compound

Contact	%
H⋯H	13.8
O⋯H/H⋯O	36.4
C⋯H/H⋯C	7.4
N⋯H/H⋯N	7.5
C⋯C	6.6
C⋯O/O⋯C	8.3
S⋯H/H⋯S	8.9
N⋯O/O⋯N	3.1
S⋯O/O⋯S	2.6
C⋯N/N⋯C	2.1
O⋯O	1.5
N⋯S/S⋯N	0.6
S⋯S	0.6
C⋯S/S⋯C	0.5
N⋯N	0.1

**Table 4:** Enrichment ratios (ER) for the title compound

Contact	ER
H⋯H	0.72
O⋯H/H⋯O	1.55
N⋯H/H⋯N	1.26
C⋯C	2.66
C⋯O/O⋯C	0.99
C⋯H/H⋯C	0.53
S⋯O/O⋯S	0.71
N⋯O/O⋯N	0.86
S⋯H/H⋯S	0.64

**Table 5:** Experimental details

Crystal data	
Chemical formula	C_10_H_7_N_3_O_4_S
*M*_r_	265.25
Crystal system, space group	Tetragonal, *I*4_1_ *c* *d*
Temperature (K)	100
*a*, *c* (Å)	17.4072 (16), 14.4881 (10)
*V* (Å^3^)	4390.0 (9)
*Z*	16
Radiation type	Mo *K*α
μ (mm^−1^)	0.31
Crystal size (mm)	0.13 × 0.03 × 0.02

Data collection	
Diffractometer	Rigaku Saturn724+ (2x2 bin mode)
Absorption correction	Multi-scan (*CrystalClear-SM Expert*; Rigaku, 2011 ▸)
*T*_min_, *T*_max_	0.543, 1.000
No. of measured, independent and observed [*I* > 2σ(*I*)] reflections	10325, 2292, 2081
*R*_int_	0.061
(sin θ/λ)_max_ (Å^−1^)	0.649

Refinement	
*R*[*F* ^2^ > 2σ(*F* ^2^)], *wR*(*F* ^2^), *S*	0.045, 0.113, 1.05
No. of reflections	2292
No. of parameters	166
No. of restraints	2
Δρ_max_, Δρ_min_ (e Å^−3^)	0.46, −0.31
Absolute structure	Flack *x* determined using 766 quotients [(*I* ^+^)−(*I* ^−^)]/[(*I* ^+^)+(*I* ^−^)] (Parsons *et al.* 2013 ▸)
Absolute structure parameter	−0.06 (6)

Computer programs: *CrystalClear-SM Expert* (Rigaku, 2011 ▸), *SHELXS97* (Sheldrick, 2008 ▸), *SHELXL2014* (Sheldrick, 2015 ▸), *ORTEP-3 for Windows* (Farrugia, 2012 ▸), *QMol* (Gans & Shalloway, 2001 ▸), *DIAMOND* (Brandenburg, 2006 ▸) and *publCIF* (Westrip, 2010 ▸).

## supplementary crystallographic information

### Crystal data

**Table:**

C_10_H_7_N_3_O_4_S	*D*_x_ = 1.605 Mg m^−^^3^
*M**_r_* = 265.25	Mo *K*α radiation, λ = 0.71073 Å
Tetragonal, *I*4_1_*c**d*	Cell parameters from 9311 reflections
*a* = 17.4072 (16) Å	θ = 3.3–27.5°
*c* = 14.4881 (10) Å	µ = 0.31 mm^−^^1^
*V* = 4390.0 (9) Å^3^	*T* = 100 K
*Z* = 16	Rod, yellow
*F*(000) = 2176	0.13 × 0.03 × 0.02 mm

### Data collection

**Table:**

Rigaku Saturn724+ (2x2 bin mode) diffractometer	2292 independent reflections
Radiation source: Rotating Anode	2081 reflections with *I* > 2σ(*I*)
Confocal monochromator	*R*_int_ = 0.061
Detector resolution: 28.5714 pixels mm^-1^	θ_max_ = 27.5°, θ_min_ = 3.3°
profile data from ω–scans	*h* = −22→22
Absorption correction: multi-scan (*CrystalClear-SM Expert*; Rigaku, 2011)	*k* = −22→13
*T*_min_ = 0.543, *T*_max_ = 1.000	*l* = −15→18
10325 measured reflections	

### Refinement

**Table:**

Refinement on *F*^2^	Hydrogen site location: mixed
Least-squares matrix: full	H-atom parameters not defined?
*R*[*F*^2^ > 2σ(*F*^2^)] = 0.045	*w* = 1/[σ^2^(*F*_o_^2^) + (0.066*P*)^2^ + 2.8065*P*] where *P* = (*F*_o_^2^ + 2*F*_c_^2^)/3
*wR*(*F*^2^) = 0.113	(Δ/σ)_max_ < 0.001
*S* = 1.05	Δρ_max_ = 0.46 e Å^−^^3^
2292 reflections	Δρ_min_ = −0.31 e Å^−^^3^
166 parameters	Absolute structure: Flack *x* determined using 766 quotients [(*I*^+^)-(*I*^-^)]/[(*I*^+^)+(*I*^-^)] (Parsons *et al.* 2013)
2 restraints	Absolute structure parameter: −0.06 (6)

### Special details

**Table:**

Geometry. All esds (except the esd in the dihedral angle between two l.s. planes) are estimated using the full covariance matrix. The cell esds are taken into account individually in the estimation of esds in distances, angles and torsion angles; correlations between esds in cell parameters are only used when they are defined by crystal symmetry. An approximate (isotropic) treatment of cell esds is used for estimating esds involving l.s. planes.

### Fractional atomic coordinates and isotropic or equivalent isotropic displacement parameters (Å^2^)

**Table:**

	*x*	*y*	*z*	*U*_iso_*/*U*_eq_	
S1	0.48085 (6)	0.10242 (6)	0.44233 (8)	0.0259 (3)	
O1	0.36361 (17)	0.16769 (16)	0.56218 (18)	0.0200 (6)	
O2	0.22698 (16)	0.39128 (15)	0.66600 (18)	0.0161 (6)	
O3	0.25495 (19)	0.40880 (19)	0.8415 (2)	0.0265 (7)	
O4	0.18400 (18)	0.51202 (19)	0.8477 (2)	0.0266 (7)	
N1	0.31555 (19)	0.25578 (19)	0.4618 (2)	0.0165 (7)	
H1N	0.319 (3)	0.278 (2)	0.4082 (17)	0.020*	
N2	0.28004 (19)	0.29467 (19)	0.5325 (2)	0.0171 (7)	
N3	0.2125 (2)	0.4568 (2)	0.8067 (2)	0.0196 (7)	
C1	0.4111 (2)	0.1666 (2)	0.4094 (3)	0.0162 (8)	
C2	0.4137 (2)	0.1857 (2)	0.3137 (3)	0.0206 (9)	
H2	0.3797	0.2195	0.2824	0.025*	
C3	0.4770 (2)	0.1443 (2)	0.2728 (3)	0.0221 (9)	
H3	0.4901	0.1482	0.2093	0.027*	
C4	0.5164 (3)	0.0990 (2)	0.3336 (3)	0.0251 (10)	
H4	0.5595	0.0686	0.3166	0.030*	
C5	0.3616 (2)	0.1960 (2)	0.4838 (3)	0.0158 (8)	
C6	0.2361 (2)	0.3499 (2)	0.5074 (3)	0.0170 (8)	
H6	0.2247	0.3576	0.4439	0.020*	
C7	0.2040 (2)	0.4004 (2)	0.5764 (3)	0.0166 (8)	
C8	0.1580 (2)	0.4633 (2)	0.5673 (3)	0.0183 (8)	
H8	0.1351	0.4815	0.5120	0.022*	
C9	0.1511 (2)	0.4962 (2)	0.6561 (3)	0.0181 (8)	
H9	0.1228	0.5406	0.6731	0.022*	
C10	0.1940 (2)	0.4502 (2)	0.7116 (3)	0.0160 (8)	

### Atomic displacement parameters (Å^2^)

**Table:**

	*U*^11^	*U*^22^	*U*^33^	*U*^12^	*U*^13^	*U*^23^
S1	0.0246 (6)	0.0270 (6)	0.0260 (5)	0.0060 (4)	0.0037 (5)	0.0022 (4)
O1	0.0246 (16)	0.0207 (15)	0.0146 (14)	0.0052 (11)	0.0020 (11)	0.0039 (11)
O2	0.0182 (14)	0.0169 (14)	0.0132 (13)	0.0042 (10)	−0.0002 (11)	−0.0039 (11)
O3	0.0289 (17)	0.0334 (18)	0.0174 (15)	0.0083 (13)	−0.0045 (13)	−0.0010 (13)
O4	0.0333 (18)	0.0288 (18)	0.0179 (15)	0.0057 (14)	0.0017 (13)	−0.0096 (13)
N1	0.0219 (17)	0.0185 (17)	0.0093 (15)	0.0042 (13)	0.0034 (13)	0.0006 (13)
N2	0.0183 (17)	0.0187 (16)	0.0142 (16)	−0.0004 (13)	−0.0009 (13)	−0.0027 (13)
N3	0.0209 (17)	0.0195 (18)	0.0183 (17)	0.0003 (13)	0.0021 (14)	−0.0034 (13)
C1	0.0161 (18)	0.0135 (18)	0.0191 (19)	−0.0022 (14)	0.0016 (15)	−0.0006 (14)
C2	0.020 (2)	0.0125 (19)	0.029 (2)	−0.0047 (14)	0.0117 (17)	−0.0102 (16)
C3	0.028 (2)	0.019 (2)	0.020 (2)	0.0015 (16)	0.0085 (17)	−0.0002 (16)
C4	0.021 (2)	0.027 (2)	0.027 (2)	0.0054 (17)	0.0089 (18)	−0.0021 (18)
C5	0.0177 (19)	0.0146 (18)	0.0151 (18)	−0.0033 (14)	−0.0004 (15)	−0.0007 (14)
C6	0.0170 (19)	0.019 (2)	0.0150 (18)	0.0014 (14)	0.0004 (14)	−0.0017 (15)
C7	0.0176 (19)	0.023 (2)	0.0096 (18)	−0.0021 (15)	−0.0001 (14)	0.0016 (15)
C8	0.018 (2)	0.022 (2)	0.0153 (19)	0.0012 (14)	0.0007 (15)	0.0013 (16)
C9	0.020 (2)	0.0170 (19)	0.0176 (19)	0.0015 (15)	0.0042 (15)	0.0017 (15)
C10	0.0156 (19)	0.0174 (18)	0.0150 (18)	−0.0003 (14)	0.0021 (14)	−0.0013 (14)

### Geometric parameters (Å, º)

**Table:**

S1—C4	1.694 (5)	C1—C5	1.472 (5)
S1—C1	1.718 (4)	C2—C3	1.444 (6)
O1—C5	1.239 (5)	C2—H2	0.9500
O2—C10	1.349 (5)	C3—C4	1.367 (6)
O2—C7	1.367 (5)	C3—H3	0.9500
O3—N3	1.225 (5)	C4—H4	0.9500
O4—N3	1.234 (4)	C6—C7	1.443 (5)
N1—C5	1.351 (5)	C6—H6	0.9500
N1—N2	1.374 (5)	C7—C8	1.364 (6)
N1—H1N	0.870 (14)	C8—C9	1.413 (6)
N2—C6	1.281 (5)	C8—H8	0.9500
N3—C10	1.419 (5)	C9—C10	1.358 (6)
C1—C2	1.427 (6)	C9—H9	0.9500
			
C4—S1—C1	91.3 (2)	S1—C4—H4	123.4
C10—O2—C7	104.6 (3)	O1—C5—N1	122.6 (4)
C5—N1—N2	118.1 (3)	O1—C5—C1	121.1 (4)
C5—N1—H1N	120 (3)	N1—C5—C1	116.3 (3)
N2—N1—H1N	119 (3)	N2—C6—C7	119.4 (3)
C6—N2—N1	115.3 (3)	N2—C6—H6	120.3
O3—N3—O4	125.1 (4)	C7—C6—H6	120.3
O3—N3—C10	118.9 (3)	O2—C7—C8	110.9 (3)
O4—N3—C10	116.0 (3)	O2—C7—C6	118.3 (3)
C2—C1—C5	130.5 (4)	C8—C7—C6	130.5 (4)
C2—C1—S1	113.5 (3)	C7—C8—C9	106.7 (4)
C5—C1—S1	115.9 (3)	C7—C8—H8	126.7
C1—C2—C3	107.8 (4)	C9—C8—H8	126.7
C1—C2—H2	126.1	C10—C9—C8	104.7 (4)
C3—C2—H2	126.1	C10—C9—H9	127.6
C4—C3—C2	114.0 (4)	C8—C9—H9	127.6
C4—C3—H3	123.0	O2—C10—C9	113.1 (3)
C2—C3—H3	123.0	O2—C10—N3	116.1 (3)
C3—C4—S1	113.3 (3)	C9—C10—N3	130.7 (4)
C3—C4—H4	123.4		
			
C5—N1—N2—C6	−178.9 (3)	C10—O2—C7—C8	−0.1 (4)
C4—S1—C1—C2	0.7 (3)	C10—O2—C7—C6	174.1 (3)
C4—S1—C1—C5	−176.2 (3)	N2—C6—C7—O2	5.4 (6)
C5—C1—C2—C3	175.6 (4)	N2—C6—C7—C8	178.2 (4)
S1—C1—C2—C3	−0.7 (4)	O2—C7—C8—C9	−0.1 (4)
C1—C2—C3—C4	0.4 (5)	C6—C7—C8—C9	−173.3 (4)
C2—C3—C4—S1	0.2 (5)	C7—C8—C9—C10	0.2 (4)
C1—S1—C4—C3	−0.5 (3)	C7—O2—C10—C9	0.2 (4)
N2—N1—C5—O1	11.6 (5)	C7—O2—C10—N3	−176.4 (3)
N2—N1—C5—C1	−167.3 (3)	C8—C9—C10—O2	−0.3 (4)
C2—C1—C5—O1	174.0 (4)	C8—C9—C10—N3	175.8 (4)
S1—C1—C5—O1	−9.8 (5)	O3—N3—C10—O2	−1.7 (5)
C2—C1—C5—N1	−7.1 (6)	O4—N3—C10—O2	178.1 (3)
S1—C1—C5—N1	169.1 (3)	O3—N3—C10—C9	−177.6 (4)
N1—N2—C6—C7	−172.1 (3)	O4—N3—C10—C9	2.1 (6)

### Hydrogen-bond geometry (Å, º)

**Table:**

*D*—H···*A*	*D*—H	H···*A*	*D*···*A*	*D*—H···*A*
N1—H1*N*···O1^i^	0.87 (3)	2.05 (3)	2.882 (4)	159 (3)
C4—H4···O4^ii^	0.95	2.42	3.293 (6)	152
C8—H8···O4^iii^	0.95	2.53	3.242 (5)	132

Symmetry codes: (i) −*y*+1/2, *x*, *z*−1/4; (ii) *x*+1/2, *y*−1/2, *z*−1/2; (iii) *x*, −*y*+1, *z*−1/2.
